# The influence of symptom severity of palliative care patients on their family caregivers

**DOI:** 10.1186/s12904-022-00918-3

**Published:** 2022-02-28

**Authors:** Inmaculada Valero-Cantero, Cristina Casals, Yolanda Carrión-Velasco, Francisco Javier Barón-López, Francisco Javier Martínez-Valero, María Ángeles Vázquez-Sánchez

**Affiliations:** 1 Puerta Blanca Clinical Management Unit, Malaga-Guadalhorce Health District, Malaga, Spain; 2 MOVE-IT Research Group and Department of Physical Education, Faculty of Education Sciences, University of Cádiz, Cádiz, Spain; 3 Instituto de Investigación E Innovación Biomédica de Cádiz (INiBICA), Hospital Universitario Puerta del Mar, Universidad de Cádiz, Cádiz, Spain; 4 La Luz Clinical Management Unit, Malaga-Guadalhorce Health District, Malaga, Spain; 5 Faculty of Health Sciences, University of Málaga, and Instute of Biomedical Research in Málaga (IBIMA), Málaga, Spain; 6 Midlothian Foot Care, Dalkeith and National Health Service, Lothian, Scotland, UK; 7 Department of Nursing, Faculty of Health Sciences, UMA REDIAS Network of Law and Artificial Intelligence Applied To Health and Biotechnology, University of Málaga, Málaga, Spain

**Keywords:** Caregivers, Palliative care, Medical oncology, Home care services, Community health nursing

## Abstract

**Background:**

This study anlyzed whether family caregivers of patients with advanced cancer suffer impaired sleep quality, increased strain, reduced quality of life or increased care burden due to the presence and heightened intensity of symptoms in the person being cared for.

**Method:**

A total of 41 patient-caregiver dyads (41 caregivers and 41 patients with advanced cancer) were recruited at six primary care centres in this cross-sectional study. Data were obtained over a seven-month period. Caregiver’s quality of sleep (Pittsburgh Sleep Quality Index), caregiver’s quality of life (Quality of Life Family Version), caregiver strain (Caregiver Strain Index), patients’ symptoms and their intensity (Edmonton Symptom Assessment System), and sociodemographic, clinical and care-related data variables were assessed. The associations were determined using non-parametric Spearman correlation.

**Results:**

Total Edmonton Symptom Assessment System was significantly related to overall score of the Pittsburgh Sleep Quality Index (*r* = 0.365, *p* = 0.028), the Caregiver Strain Index (*r* = 0.45, *p* = 0.005) and total Quality of Life Family Version (*r* = 0.432, *p* = 0.009), but not to the duration of daily care (*r* = -0.152, *p* = 0.377).

**Conclusions:**

Family caregivers for patients with advanced cancer suffer negative consequences from the presence and intensity of these patients’ symptoms. Therefore, optimising the control of symptoms would benefit not only the patients but also their caregivers. Thus, interventions should be designed to improve the outcomes of patient-caregiver dyads in such cases.

## Background

Rising numbers of patients with advanced cancer are receiving home palliative care, due to the increased prevalence of this disease [1] and to the recognised benefits of early palliative care [2, 3] in terms of patient satisfaction and the alleviation of the changeable and frequently severe symptoms presented [4–7].

Given the characteristics and often worsening nature of their symptoms, many patients with advanced cancer require quality palliative health care in the home, which in most cases is provided by a family caregiver, in collaboration with the health system [8–10]. In home palliative care, these family caregivers are usually relatives of the patient, most commonly spouse, parent or son/daughter, although other family members or friends sometimes perform this role, for which no financial compensation is obtained [11, 12].

Caregivers of patients with advanced cancer can be affected both physically and psychologically [13] and be subjected to considerable demands on their time, physical energy and mental resources [14]. The most frequent disorders experienced by such caregivers involve their mental health, in areas such as depressed mood and anxiety [15, 16]. Others include fatigue [17, 18], impaired sleep [19, 20], caregiver overload and overall reduced quality of life [21–24].

These disorders are well documented; however, their relation with the patient’s condition has received less research attention, and the few studies that have investigated this question have reported widely varying conclusions [25–27]. Thus, the main aim of this study is to determine whether the presence and intensity of the symptoms of patients with advanced cancer have a negative impact on the family caregiver, in terms of sleep quality, strain, quality of life and care burden.

## Methods

### Eligibility criteria and sampling

For this descriptive cross-sectional study, the patient-caregiver dyads were recruited at six primary care centres in the Málaga-Guadalhorce Health District (Málaga, Spain).

**Fig. 1:**
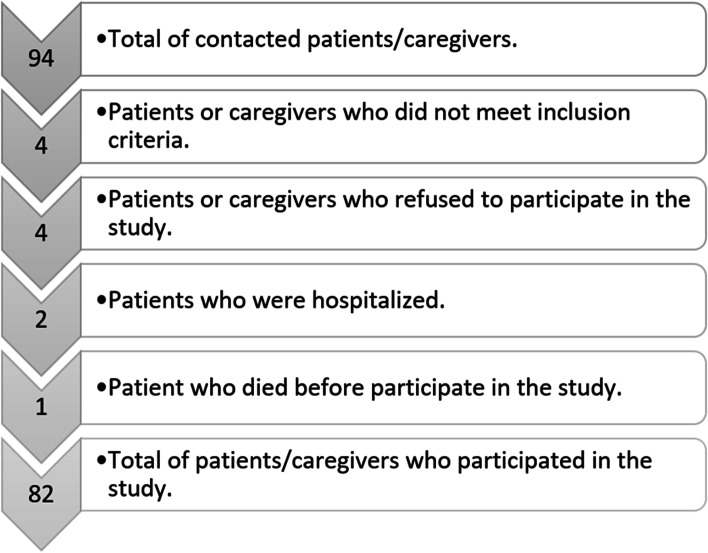
Flowchart of the patients included in the study

The study sample was drawn from the lists of cancer patients recorded under the Palliative Care Assistance Process. The following inclusion criteria were applied for participation: (1) Cancer patients in home palliative care who have a family caregiver; (2) Both patient and caregiver are aged ≥ 18 years; (3) Both patient and caregiver give signed informed consent to participate. Excluded from the study were (1) Patients with advanced disease, whose life expectancy was only a few days; (2) Patients with advanced stage dementia or psychological disorders making them incapable of taking rational decisions. The selection of participants is detailed in Fig. 1.

### Measures

The following study variables were considered:

#### Sociodemographic, clinical and care-related data

Caregivers: age, sex, marital status, education, paid employment, daily hours dedicated to care, relationship with the patient. These variables were indicated by the caregiver in a personal interview.

Patients in palliative care: age, sex, marital status, education, total time in palliative care and type of cancer. Total time of palliative care was estimated according to the date in which oncology referred the patient to palliative care, this data is available in the patient’s health record.

#### Caregiver’s quality of sleep

The caregiver’s quality of sleep was determined using the Pittsburgh Sleep Quality Index (PSQI) [28], which evaluates seven sleep domains: subjective quality, latency, duration, efficiency, sleep disorders, use of medications for sleep and daytime dysfunction during the past month. The sum of the scores obtained in each of the seven partial components generates a total score, ranging from 0–21. A total score > 5 indicates poor sleep quality.

#### Caregiver’s quality of life

The caregiver’s quality of life (QoL) was evaluated using the Quality of Life Family Version (FQOL) instrument [29], which is commonly used to assess the QoL of caregivers of patients with chronic disease. The FQOL consists of 35 items, scored from 1 to 4, assessing physical, psychological, spiritual and social aspects of QoL. The total score obtained ranges from 0 to 100, with 100 being the best possible QoL.

#### Caregiver strain

Caregiver strain was determined using the Caregiver Strain Index (CSI) questionnaire [30], which consists of 13 dichotomous (Yes–No) items. Each affirmative answer scores 1, and the total score obtained ranges from 0 to 13 points. A total score of ≥ 7 suggests a high level of strain.

#### Patients’ symptoms and their intensity

The patients’ symptoms and their intensity were measured using the Edmonton Symptom Assessment System (ESAS) scale [31, 32]. This instrument evaluates the presence and intensity of the following symptoms, during a specific period: pain, fatigue, nausea, depression, anxiety, drowsiness, dyspnoea, appetite, reduced wellbeing and sleep alterations. The intensity of the symptoms is scored from 0 to 10, with 0 meaning absence of the symptom and 10 its greatest possible severity. The total score obtained ranges from 0 to100.

All mentioned questionnaires were applied in caregivers and patient in the validated Spanish version.

### Data collection

The study sample consisted of 41 patients and the corresponding 41 caregivers, who were recruited during the period June-December 2020, according to the following procedure. After confirming that both the patient and his/her family caregiver met the inclusion criteria, they were fully informed about the study (both orally and in writing). If both parties agreed to participate, they were then asked to sign the informed consent form. Subsequently, the caregiver-patient dyads were interviewed in their home by a nurse collaborating with the study and asked to complete the questionnaires.

### Statistical methods

The characteristics of the participants are presented as mean values (± standard deviation (SD)) for the quantitative variables, and as absolute frequencies (n) and percentages (%) for the categorical ones. The associations between total ESAS and ‘hours per day dedicated to care’, total PSQI, total FQOL and CSI were determined using non-parametric Spearman correlation. All statistical analyses were conducted using Statistical Package for Social Sciences (SPSS) 22 software, and *p* ≤ 0.05 was considered significant.

## Results

### Sociodemographic, clinical and care-related characteristics of a) patients with advanced cancer; b) their caregivers

Initial contact was made with 94 potential participants. Of these, eight did not meet the inclusion criteria, four caregivers and two patients declined to participate, and six patients died before the interview could be held. Table 1 shows the sociodemographic characteristics of the caregivers, and Table 2, the corresponding data for the patients.

**Table 1:** Demographic characteristics of the family caregiver (*n* = 41)

**Age:** mean 63.88; SD 8.29		
**Daily hours’ care:** mean 18.83; SD 6.77		
	**N**	**%**
**Gender**		
Female	36	87.8
Male	5	12.2
**Relationship with patient**		
Spouse	24	58.5
Son/Daughter	14	34.1
Sister	2	4.9
Daughter-in-law	1	2.4
**Marital status**		
Married	30	73.2
Divorced	5	12.2
Single	4	9.8
Widowed	2	4.9
**Children**		
Two	25	61
None	8	19.5
One	7	17.1
Three	1	2.4
**Education**		
Primary	21	51.2
Secondary	12	29.3
No formal education	7	17.1
University	1	2.4
**Employment status**		
Never in paid employment	24	58.5
Currently in paid employment	8	19.5
Retired	7	17.1
Unemployed	2	4.9

**Table 2:** Demographic characteristics of the patient (*n* = 41)

**Age (years):** Mean 73.57; SD 11.80		
**Duration of palliative care (months):** Mean 4.54; SD 5.99		
	**N**	**%**
**Gender**		
Female	23	56
Male	18	44
**Marital status**		
Married	21	73.2
Divorced	15	12.2
Single	3	9.8
Widowed	2	4.9
**Education**		
Primary	18	51.2
Secondary	9	29.3
No formal education	9	17.1
University	5	2.4
**Type of cancer**		
Colorectal cancer	9	21.95
Lung cancer	6	14.63
Breast cancer	6	14.63
Prostate cancer	5	12.20
Oropharyngeal cancer	4	9.76
Myelodysplasia	4	9.76
Liver cancer	3	7.32
Lymphoma	2	4.88
Brain cancer	1	2.44
Pancreatic cancer	1	2.44

### Descriptive data for the study variables

The overall mean PSQI for the caregivers was 7.66 (SD: 3.81). A poor quality of sleep (PSQI > 5) was reported by 31 caregivers, or 75.6% of the sample. The following mean FQOL scores were obtained for the caregivers: physical aspects 38.04 (SD: 19.73), psychological aspects 52.67 (SD: 11.08), spiritual aspects 65.73 (SD: 14.7), social aspects 39.74 (SD: 18.46), and total FQOL 52.75 (SD: 12.11). The mean CSI result was 6.8 (SD: 3.03). A score of ≥ 7, suggesting a high level of effort, was recorded for 23 caregivers (56.1%). Table 3 shows the patients’ symptom severity, according to the ESAS scores obtained.

**Table 3:** Edmonton Symptom Assessment System

**Symptoms**	*Mean*	*SD*
Pain	4.22	3.08
Fatigue	5.78	2.84
Nausea	0.94	1.98
Depression	4.64	3.31
Anxiety	3.56	3.55
Drowsiness	4.83	3.19
Dyspnoea	2.08	2.94
Appetite	3.28	3.14
Reduced wellbeing	5.86	2.17
Sleep	4.42	3.26
**Total ESAS symptoms**	39.61	15.48

The CSI was significantly related to the total FQOL (*r* = -0.610, *p* < 0.001), and with the physical (*r* = -0.472, *p* = 0.002), psychological (*r* = -0.418, *p* = 0.007) and sociological (*r* = -0.550 *p* < 0.001) scales; the association with the spiritual subscale did not reach statistical significance (*r* = -0.297, *p* = 0.063). The greater the effort of the caregiver, the worse quality of life.

### Associations between ESAS and caregiving parameters

Total ESAS symptoms were significantly related to overall Pittsburgh score (*r* = 0.365, *p* = 0.028), the CSI (*r* = 0.45, *p* = 0.005) and total FQOL (*r* = 0.432, *p* = 0.009), but not to the duration of daily care (*r* = -0.152, *p* = 0.377). Table 4 presents bivariate correlations of ESAS and caregiving parameters.

**Table 4:** Bivariate associations

	**CSI**	**ESAS**	**PSQI**	**FQOL**
**CSI**	-	0.455	0.538	-0.592
* P value*	-	0.005	< 0.001	< 0.001
**ESAS**	0.455	-	0.365	-0.432
* P value*	0.005	-	0.028	0.009
**PSQI**	0.538	0.365	-	-0.387
* P value*	< 0.001	0.28	-	0.015

There were no association between the time caring, or the time in palliative care and the outcome variables in the caregiver, nor with the patient’s symptom scale.

## Discussion

In our study sample, most of the caregivers were women, confirming the pattern observed in previous studies [12, 33]. However, among the patients, the sexes were more evenly balanced [34]. In most cases, women continue to play the role of family caregiver. In our sample, all of the caregivers were family members, usually spouses or children [35] and on average dedicated extremely long hours to this task, exceeding 18 h a day [12].

When this study was conducted, the mean duration of palliative care for these patients was greater than four months, which suggests that in most cases early referral takes place, which is generally considered to reflect good patient care [3]. The types of cancer suffered by the patients in this study fitted the global pattern reported in this respect [36].

Among the patients in our study, the symptoms presented with greatest intensity were reduced wellbeing, fatigue, daytime sleepiness, depression, and simultaneous sleepiness and pain, which is in line with previous research findings [5, 7]. The mean scores obtained for the severity of these symptoms (4–6) reflect a moderate degree of intensity [32]. Whilst open to improvement, this level of symptoms is lower than that found in patients with similar clinical conditions who are not receiving palliative care [5].

Most of the caregivers were affected by strain, which was reflected in each of the variables studied. Over 75% of the caregivers reported suffering sleep disorders, a result that is similar to previous research findings [12, 20]. Such alterations provoke or aggravate problems such as anxiety, diabetes, obesity, heart disease and stroke [37–39].

A similar negative impact was reflected in the caregiver strain index, the score for which was significantly high for over 56% of the caregivers in our sample. This burden has been associated with increased anxiety and depression in previous literature [34, 35]. The caregiver strain compromised the total quality of life, also the physical, psychological and sociological scales,however, the association with the spiritual subscale did not reach statistical significance. Reflecting these consequences, the results for the caregivers’ overall quality of life, with an overall mean score of 52%, were also unsatisfactory [21].

These findings highlight the novel contribution made by our study, as little previous research has been undertaken regarding the association of symptoms of patients with advanced cancer on their caregivers’ quality of life. We show that a greater overall severity of symptoms suffered by patients with advanced cancer is related to worse quality of sleep for the caregiver. To our knowledge, only one previous study has considered such a relationship; in this case, the patient’s feeling of distress was directly associated with impairment of the caregiver’s sleep [40].

We also find that the greater severity of the patient’s symptoms is directly related to caregiver strain, which corroborates the findings of several prior studies in this regard [24, 41, 42]. Finally, the increased intensity of the patient’s symptoms is related to a poorer overall quality of life for the caregiver.

Overall, our study findings highlight the negative impact produced on the caregiver by the increasing severity of the patient’s condition, and underline the need to pay specific attention to helping the family caregivers of patients with advanced cancer receiving home palliative treatment. Our analysis of the relationship between the study variables considered for the patient-caregiver dyad shows that the caregiver benefits, indirectly, when the patient’s symptoms are alleviated.

This study has certain limitations that must be acknowledged. Firstly, the results obtained should be interpreted taking into account the cross-sectional nature of the study, which means that causality cannot be affirmed. Moreover, variables other than those included in the study may also influence the status of the family caregiver. Finally, the reliability of the analysis might be affected by the limited sample size of the caregiver-patient dyad in our analysis; this restriction arose from the difficulty encountered in recruiting suitable participants for the study, which in each case required the agreement of both the caregiver and the patient.

Further research should be conducted to design and develop interventions to improve the situation of patient-caregiver dyads when palliative home care is provided for patients with advanced cancer.

## Conclusions

Family caregivers for patients with advanced cancer suffer negative consequences from the presence and intensity of these patients’ symptoms. Therefore, optimising the control of symptoms would benefit not only the patients but also their caregivers.

## Data Availability

The datasets used and/or analysed during the current study available from the corresponding author on reasonable request.

## Abbreviations

CSI: Caregiver Strain Index
ESAS: Edmonton Symptom Assessment System
FQOL: Quality of Life Family Version
PSQI: Pittsburgh Sleep Quality Index
QoL: Quality of life
SD: Standard deviation
SPSS: Statistical Package for Social Sciences
